# Local phytochrome signalling limits root growth in light by repressing auxin biosynthesis

**DOI:** 10.1093/jxb/erad163

**Published:** 2023-05-04

**Authors:** Kiki Spaninks, Remko Offringa

**Affiliations:** Plant Developmental Genetics, Institute of Biology Leiden, Leiden University, Sylviusweg 72, 2333 BE, Leiden, Netherlands; Plant Developmental Genetics, Institute of Biology Leiden, Leiden University, Sylviusweg 72, 2333 BE, Leiden, Netherlands; University of Maryland, USA

**Keywords:** Arabidopsis, auxin biosynthesis, phytochrome signalling, root growth, tomato

## Abstract

In nature, plant shoots are exposed to light whereas the roots grow in relative darkness. Surprisingly, many root studies rely on *in vitro* systems that leave the roots exposed to light whilst ignoring the possible effects of this light on root development. Here, we investigated how direct root illumination affects root growth and development in Arabidopsis and tomato. Our results show that in light-grown Arabidopsis roots, activation of local phytochrome A and B by far-red or red light inhibits respectively PHYTOCHROME INTERACTING FACTORS 1 or 4, resulting in decreased *YUCCA4* and *YUCCA6* expression. As a result, auxin levels in the root apex become suboptimal, ultimately resulting in reduced growth of light-grown roots. These findings highlight once more the importance of using *in vitro* systems where roots are grown in darkness for studies that focus on root system architecture. Moreover, we show that the response and components of this mechanism are conserved in tomato roots, thus indicating its importance for horticulture as well. Our findings open up new research possibilities to investigate the importance of light-induced root growth inhibition for plant development, possibly by exploring putative correlations with responses to other abiotic signals, such as temperature, gravity, touch, or salt stress.

## Teaser

In widely used *in vitro*plant growth systems, illumination of roots leads to suboptimal auxin levels in root apical meristem, limiting root growth in response to red and far-red light.

## Introduction

Light is an essential energy source for life on earth. Aside from driving photosynthesis in cyanobacteria and plants, light also acts as an environmental cue that regulates almost all aspects of plant growth and development. Perception of light by photoreceptors initiates a variety of physiological responses that are collectively referred to as photomorphogenesis (Arsovski *et al.*, 2012). The blue light photoreceptor families of cryptochromes (CRYs), phototropins (PHOTs), and Zeitlupes act together with the red (R)/far-red (FR)-sensitive family of phytochromes (PHYs) to regulate developmental processes ranging from germination to flowering, often by influencing hormonal pathways (de Wit *et al.*, 2016). Generally, only the plant shoot is considered when light perception is discussed, as in nature plant roots grow in a relatively dark environment. However, root morphology and development are greatly influenced by light (Lee *et al.*, 2017). When roots are covered by soil or sand, not only is the light intensity greatly reduced, but spectral changes may occur as well due to differential penetration of light of certain wavelengths (Mandoli *et al.*, 1990). Photoreceptors regulate root development either by detecting light in the shoot and inducing transmission of mobile signalling molecules, or by perceiving direct or stem-piped light in the roots (Kiss *et al.*, 2003; Lejay *et al.*, 2008; Sassi *et al.*, 2012; Chen *et al.*, 2016; Lee *et al.*, 2016). A healthy root system is vital for plants for the absorption of water and nutrients, for mechanical support, and as a sink organ (Petricka *et al.*, 2012). Root-localized light perception is physiologically relevant when growing plants *in vitro* or in aeroponic systems. Therefore, elucidation of the local light perception and signalling pathways in the roots is particularly important for studies that focus on root system architecture (RSA) and that have been conducted in *in vitro* systems where the plant roots are exposed to light. Excluding the effect of light, while using light-grown root (LGR) systems in these studies, might result in inadequate predictive models for RSA phenotypes. For example, an immediate and strong outburst of reactive oxygen species (ROS) has been observed in roots grown in LGR conditions, which might influence the overall RSA (Yokawa *et al.*, 2011). To avoid such stresses, and their adverse effects on the RSA, a dark-grown root (DGR) system, such as the D-root system, should be used for future RSA studies (Silva-Navas *et al.*, 2015). Also in horticulture, where plants are often grown in aeroponic systems or on light-transmittable substrates, such as glass wool, the unintended LGR conditions may influence the growth and development of crop plants. Although crop breeding programmes mainly focus on shoot-related phenotypes, changes in RSA might improve crop tolerance to a range of abiotic stresses including drought, salinity, and nutrient limitations (Koevoets *et al.*, 2016).

Here we show that when Arabidopsis seedlings are grown in the DGR condition, the bHLH transcription factors PHYTOCHROME INTERACTING FACTOR 1 (PIF1) and PIF4 promote local auxin biosynthesis through up-regulation of *YUCCA4* (*YUC4*) and *YUC6* genes, which results in close-to-optimal auxin levels in the RAM, and thus in normal root development. However, in the LGR condition, FR or R light activation of respectively PHYA and PHYB triggers the targeted degradation of these PIFs, resulting in reduced expression of *YUC4* and *YUC6*, and ultimately in shorter roots due to suboptimal auxin levels in the RAM. In addition to the identification of this molecular mechanism, we show that the LGR response and components of this pathway are conserved between Arabidopsis and the horticultural crop tomato (*Solanum lycopersicum*).

## Materials and methods

### Growth conditions and light treatments

In all experiments, seedlings were grown at a 16 h photoperiod, under white TL lights with a measured photon flux density of 150 ± 10 μmol m^−2^ s^−1^, a temperature of 21 °C and 50% relative humidity. Two different light treatments were included: (i) seedlings were grown completely exposed to light (light-grown roots or LGR); or (ii) seedlings were grown in a more ‘natural’ light environment with shoots exposed to light and roots shielded from light using black paper covers (dark-grown roots or DGR) (Supplementary Fig. S1) (based on Silva-Navas *et al.*, 2015).

### Plant lines and seed germination

Wild-type seedlings of Arabidopsis and *Solanum lycopersicum* (tomato) were used as controls in this study. For Arabidopsis two ecotypes were included: Columbia (Col-0) and Landsberg *erecta* (L*er*). For tomato, the Moneymaker (MM) cultivar and the commercial hybrid line Foundation (FO) were used. All Arabidopsis and tomato mutants and reporter lines that were used are listed in Supplementary Table S1. Arabidopsis single mutants *phyA*, *phyB*, *pif1*, and *pif4* (all in Col-0 background) have been described before (Mayfield *et al.*, 2007; Ruckle *et al.*, 2007; Leivar *et al.*, 2008; Stephenson *et al.*, 2009), and were crossed with *pDR5::GFP* to monitor auxin responses in these lines. Prior to the experiments all mutant lines and crosses were genotyped using the primers listed in Supplementary Table S2 and if required CAPS/PCR-RFLP markers described in Supplementary Table S3 (Nam *et al.*, 1989; Konieczny and Ausubel, 1993). Arabidopsis and tomato seeds were surface-sterilized by incubating for 1 min in 70% ethanol and 10 min in a 2-fold diluted commercial bleach solution (1% chlorine). Subsequently the seeds were washed five times with sterile water. Arabidopsis seeds were stratified for 5 d at 4 °C in darkness and germinated on square plates (cat. no. 688102, Greiner Bio-One) containing ‘MA medium’ (that of Masson and Paszkowski, 1992) supplemented with 1% (w/v) sucrose and 0.8% (w/v) Daishin agar. Arabidopsis seeds were germinated by placing the plates vertically in the two light conditions described above. Sterile tomato seeds were placed on sterilized, wet filter paper (cat. no. 1001325, Whatman) using forceps and were germinated in darkness at 21 °C for 5 d. Geminated seeds were moved from the filters to square plates containing solid MA medium and placed vertically in the two light conditions described above.

### *In vitro* analysis of seedling growth

At 7 days after germination (DAG), Arabidopsis seedlings were photographed, and primary root length and hypocotyl length were measured. The shoot/root ratio was calculated based on these measurements. Tomato seedlings were photographed at 5 DAG for primary root length measurements. To monitor the response of Arabidopsis seedlings to exogenous auxin, 4-day-old seedlings were transferred to square plates containing MA medium supplemented with 0, 5, 10, 20, 30, 40, or 50 nM 1-naphthaleneacetic acid (NAA). The increase in primary root length between 0 and 6 d after NAA treatment was measured. At 6 d after NAA treatment, *pDR5::GFP* seedlings were analysed under the confocal microscope. To analyse root-localized versus shoot-localized phytochrome functions, 4-day-old Arabidopsis seedlings grown in the LGR condition were grafted as described previously (Marsch-Martínez *et al.*, 2013) in the following combinations: wild type–wild type (positive control); mutant–mutant (negative control); wild type–mutant (mutation only present in roots), and mutant–wild type (mutation only present in shoots). At 5 d after grafting, successful grafts were photographed to measure the post-grafting increase in primary root growth and analysed under the confocal microscope. To analyse the response of roots to light quality, the roots were covered with red translucent plastic (red light-grown roots, RGR) or blue translucent plastic (blue light-grown roots, BGR). To avoid any additional effects of decreased light intensity, LGR seedlings were wrapped with white translucent plastic in this experiment. The primary root length was measured after 7 d of growth under coloured plastic. All measurements were performed with ImageJ (Fiji; Schindelin *et al.*, 2012).

### Microscopy analysis

For confocal images of Arabidopsis roots, 7-day-old seedlings were stained with 10 µg ml^−1^ propidium iodide (PI) for 5 min and then mounted onto a glass slide in water with a cover slip. To visualize *pDR5::GFP* and PI staining in root tips, a Zeiss LSM5 Exciter/AxioImager equipped with a ×40 oil objective and respectively a 488 nm argon laser and a 505–530 nm band pass filter or 600 nm long pass filter was used. For images of tomato roots, 5-day-old seedlings were mounted on a glass slide and imaged with a Leica MZ16FA equipped with a Leica DFC420C camera. Yellow fluorescent protein (YFP) fluorescence was detected using a 510/520 nm excitation filter and a 560/540 nm emission filter. The corrected total cell fluorescence method (McCloy *et al.*, 2014) was slightly adjusted to quantify the corrected total fluorescence (CTF) in the root apex, where CTF=integrated density (sum of all pixel intensities)–(area of root apex×mean fluorescence of background readings). All CTF measurements were performed in ImageJ (Fiji) and are expressed in arbitrary units (A.U.).

### RNA extraction and qRT-PCR

Root tips of 7-day-old Arabidopsis seedlings or 5-day-old tomato seedlings were pooled (±80 per RNA sample), frozen in liquid nitrogen, and ground with a TissueLyser II (cat. no. 85300, Qiagen). Total RNA was extracted from the ground tissue using an RNeasy Plant Mini kit (cat. no. 74904, Qiagen), and used for first strand cDNA synthesis with the RevertAid First Strand cDNA Synthesis kit (cat. no. K1621, Thermo Fisher Scientific). For qRT-PCR, the cDNA was diluted 10× and used with TB Green Premix Ex Taq II (Tli RNase H Plus) (cat. no. RR820B, Takara) and the CFX96 TouchTM Real-Time PCR Detection System (cat. no. 1855196, Bio-Rad). *C*_T_ values were obtained using Bio-Rad CFX manager 3.1. Normalization was done according to the ΔΔ*C*_T_ method with *PP2A-3* (At2g42500) and β-tubulin-6 (At5g12250) as reference genes for Arabidopsis, and *TIP41* (Solyc10g049850) and *SAND* (Solyc03g115810) as reference genes for tomato (Pfaffl, 2001). All primers that were used for qRT-PCR are listed in Supplementary Table S2.

### Linear regression analysis

The correlation coefficient (*r*) was calculated using the equation:


$$\begin{array}{l} r=\frac{\sum \left(x_{i}-\bar{x}\right)\left(y_{i}-\bar{y}\right)}{\sqrt{\sum {\left(x_{i}-\bar{x}\right)}^{2}\;\sum {\left(y_{i}-\bar{y}\right)}^{2}}} \end{array}$$

where *x* represents the NAA concentration, and *y* represents the *pDR5::GFP* signal. To calculate the linear regression coefficients *a* (*y*-intercept) and *b* (slope), the following equations were used:


$$\begin{array}{l} a=\bar{y}-b\bar{x}\;\;\;\;\;\;\;\;\;\;\;\;\;\;\;\;\;\;\;\;\;\;\;\;b=\frac{\sigma \left(x,y\right)}{\sigma \left(x\right)} \end{array}$$

where σ(*x*, *y*) represents the covariance of *x* and *y*, and σ(*x*) represents the variance of *x*.

### Statistical analysis and figures

All phenotyping and microscopy experiments were performed with 20 or 30 biologically independent seedlings for tomato or Arabidopsis, respectively. In experiments that included only wild-type seedlings, the LGR condition was compared with the DGR condition using a two-sided Student’s *t*-test. Experiments that included NAA treatments or wild type versus mutant comparisons were statistically analysed using a one-way ANOVA followed by Tukey’s honestly significant different (HSD) post-hoc test. In qRT-PCR experiments, three biological replicates (RNA isolated from ±80 root tips) were included, with three technical replicates each. For each plant line, normalized levels of gene expression in the LGR condition were compared with the DGR condition using a two-sided Student’s *t*-test, or the LGR/DGR ratio was compared between wild type and mutants using a one-way ANOVA followed by Tukey’s HSD post-hoc test. For the linear regression analysis, regression coefficient *b* of the LGR condition was compared with regression coefficient *b* of the DGR condition as previously described (Andrade and Estévez-Pérez, 2014). All measurements were plotted on graphs using GraphPad Prism 5 software. All photographs were taken with a Nikon D5300 camera and edited in ImageJ (Fiji). Final figures were assembled using Microsoft PowerPoint.

## Results

### Cell growth in the proximal root meristem is decreased in light-grown roots

**Fig. 1.**
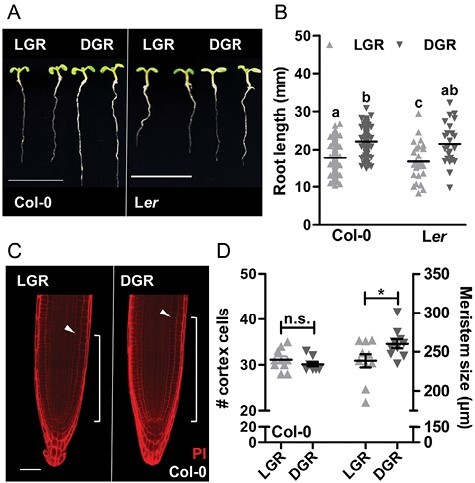
Cell growth in the proximal meristem is decreased in light-grown roots. (A) Representative 7-day-old Arabidopsis seedlings of ecotypes Columbia (Col-0) and Landsberg *erecta* (L*er*) grown in the light-grown roots (LGR) or the dark-grown roots (DGR) condition. For presentation purposes, seedlings were transferred to black agarose plates before photographing. (B) Quantification of the primary root length of 7-day-old Col-0 and L*er* seedlings grown in the LGR or DGR condition. (C) Confocal images of Col-0 root tips that were stained with propidium iodide (PI). Arrowheads indicate the end of the proximal meristem and white brackets indicate the meristem size. (D) Quantification of the proximal meristem size as number of cortex cells (left) or in µm (right) of Col-0 seedlings grown in the LGR or DGR condition. Primary root lengths in (B) were compared using one-way ANOVA followed by Tukey’s test. Lowercase letters indicate statistically different values, *P*<0.05. The LGR condition in (D) was compared with the DGR condition using a two-sided Student’s *t*-test (*P<0.05, n.s., not significant). Scale bars indicate 1 cm in (A) and 50 µm in (C). In (B) (*n*=30) and (D) (*n*=20) the horizontal line indicates the mean, error bars represent standard error of the mean (for some not visible due to limited variation), and triangles indicate values of biologically independent observations. Similar results were obtained from three (A, B), or from two (C, D) independent experiments.

Arabidopsis seedlings of ecotypes Columbia (Col-0) and Landsberg *erecta* (L*er*) were grown in the LGR or DGR condition for 7 d. Seedlings of both ecotypes showed significantly shorter roots in the LGR condition compared with the DGR condition (Fig. 1A, B), similar to previously published data (Silva-Navas *et al.*, 2015). Interestingly, hypocotyls of LGR seedlings were also significantly shorter than those of DGR seedlings (Fig. 1A; Supplementary Fig. S2A). However, since the shoot/root ratio of LGR seedlings was significantly higher than that of DGR seedlings (Supplementary Fig. S2B), we conclude that root growth inhibition in the LGR condition is independent of reduced hypocotyl growth. Root growth depends on the balance between cell proliferation and cell expansion. In general, the number and size of cortical cells in the proximal meristem of the root apex determines root length (Baskin, 2013; Aceves-García *et al.*, 2016). PI staining and imaging by confocal microscopy detected no significant differences in the number of cortical cells between root tips of LGR and DGR seedlings, whereas the proximal meristem size (in µm) was significantly smaller in LGR seedlings (Fig. 1C, D). These data showed that direct illumination of roots results in a reduced cell growth in the proximal meristem of the root apex, ultimately leading to a shorter primary root.

### Reduced growth of light-grown roots correlates with a decrease in local auxin biosynthesis in the root apical meristem

**Fig. 2.**
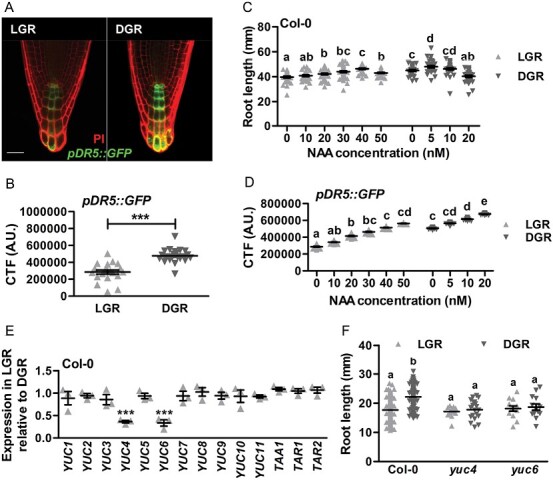
Growth inhibition of roots by light is caused by a decrease in local auxin biosynthesis in the root apical meristem (RAM). (A) Confocal images of the RAM of 7-day-old *pDR5::GFP* (green signal) seedlings grown in the LGR or the DGR condition. The roots were stained with propidium iodide (PI, red signal). (B) Quantification of the corrected total fluorescence (CTF) of the RAM. (C, D) Quantification of the primary root length of Col-0 seedlings (C) and the CTF of *pDR5::GFP* seedlings (D) grown in the LGR or DGR condition on medium containing different concentrations of 1-naphthaleneacetic acid (NAA). (E) Quantitative RT-PCR analysis of *YUC1-11*, *TAA1*, *TAR1*, and *TAR2* expression in the RAM of 7-day-old Col-0 seedlings that were grown in the LGR condition, relative to gene expression levels of seedlings grown in the DGR condition. (F) Quantification of the primary root length of 7-day-old Col-0, *yuc4*, and *yuc6* seedlings grown in the LGR or DGR condition. In (B, E), the LGR condition was compared with the DGR condition using a two-sided Student’s *t*-test (***P<0.001). In (C, D, F), NAA concentrations and primary root lengths were compared using a one-way ANOVA followed by Tukey’s test. Lowercase letters indicate statistically different values, *P*<0.05. The scale bar indicates 50 µm in (A). In (B) (*n*=20), (C, D, F) (*n*=30) and (E) (*n*=3), the horizontal line indicates the mean, error bars represent standard error of the mean (for some not visible due to limited variation), and triangles indicate values of biologically independent observations. Similar results were obtained from two (A, B) or from three (C–F) independent experiments.

As a key regulator of root growth and development, auxin might be the driving force behind cortex cell growth in the DGR condition. Confocal analysis of the *pDR5::GFP* auxin response reporter in Arabidopsis Col-0 seedlings showed a significant reduction of the green fluorescent protein (GFP) signal in the RAM of LGR seedlings, compared with DGR seedlings (Fig. 2A, B), suggesting that light inhibits the auxin response in the RAM. To investigate this, wild-type Col-0 seedlings were grown on medium supplemented with 1-naphthaleneacetic acid (NAA) concentrations varying between 0 and 50 nM. In LGR seedlings, NAA concentrations up to 40 nM maximized root growth, whereas addition of 50 nM NAA reduced root growth (Fig. 2C). In contrast, for DGR seedlings the addition of 5 nM NAA maximized root growth, whereas 20 nM NAA resulted in clear root growth inhibition. The 8-fold increase in NAA concentration for optimal root growth of LGR seedlings was in line with the reduced *DR5::GFP* expression. NAA treatment of LGR- and DGR-grown *pDR5::GFP* seedlings showed a strong correlation between enhanced reporter gene expression and primary root growth with increasing NAA concentrations, and that expression in DGR RAMs was always significantly higher compared with LGR RAMs (Fig. 2D). This correlation was linear with a statistically indistinguishable regression coefficient *b* (Supplementary Table S4), indicating that the reduced auxin response in LGR RAMs was caused by lower endogenous auxin levels, rather than a reduced auxin responsiveness. Expression analysis of the auxin biosynthesis genes *YUC1-11*, *TAA1*, *TAR1*, and *TAR2* in LGR or DGR RAMs by qRT-PCR showed that *YUC4* and *YUC6* expression was significantly lower in LGR compared with DGR seedlings (Fig. 2E). Moreover, the dark-induced enhancement of root growth was lost in *yuc4* and *yuc6* mutant seedlings grown in the DGR condition (Fig. 2F). In contrast, mutants of important auxin influx and efflux carriers remained sensitive to the different light conditions, suggesting that auxin transport is not affected in LGR seedlings (Supplementary Fig. S3A). Altogether, these experiments indicated that lower *YUC4* and *YUC6* expression in the RAM of LGR seedlings causes a reduction in local auxin biosynthesis that ultimately leads to shorter roots.

### Root-localized PHYA and PHYB mediate light-induced inhibition of root growth

**Fig. 3.**
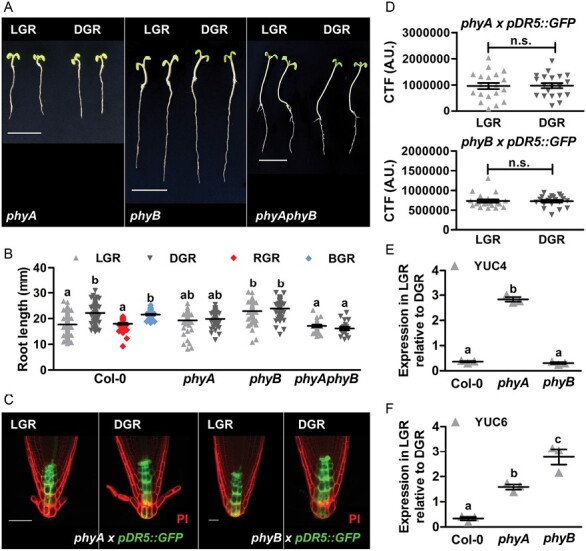
PHYA and PHYB trigger root growth inhibition in response to light. (A) Representative 7-day-old *phy* mutant seedlings grown in the LGR or the DGR condition. For presentation purposes, seedlings were transferred to black agarose plates before photographing. (B) Quantification of the primary root length of 7-day-old Col-0 seedlings grown in the LGR, DGR, red light-grown root (RGR) or blue light-grown root (BGR) condition, and *phy* seedlings grown in the LGR or DGR condition. (C) Confocal images of the root apical meristem (RAM) of *phyA × pDR5::GFP* and *phyB × pDR5::GFP* (green signal) seedlings grown in the LGR or DGR condition. Root tips were stained with propidium iodide (PI, red signal). (D) Quantification of the corrected total fluorescence (CTF) of the RAM of *phyA × pDR5::GFP* and *phyB × pDR5::GFP* seedlings. (E, F) Quantitative RT-PCR analysis of *YUC4* (E) and *YUC6* (F) expression in the RAM of 7-day-old Col-0, *phyA*, and *phyB* seedlings that were grown in the LGR condition, relative to gene expression levels in the RAM of seedlings grown in the DGR condition. In (B, E, F) primary root lengths and relative gene expression were compared using a one-way ANOVA followed by Tukey’s test. Lowercase letters indicate statistically different values, *P*<0.05. The LGR condition in (D) was compared with the DGR condition using a two-sided Student’s *t*-test (n.s., not significant). Scale bars indicate 1 cm in (A) and 50 µm in (C). In (B) (*n*=30), (D) (*n*=20) and (E, F) (*n*=3), the horizontal line indicates the mean, error bars represent standard error of the mean (for some not visible due to limited variation), and triangles indicate values of biologically independent observations. Similar results were obtained from three (A, B and E, F), or from two (C, D) independent experiments.

**Fig. 4.**
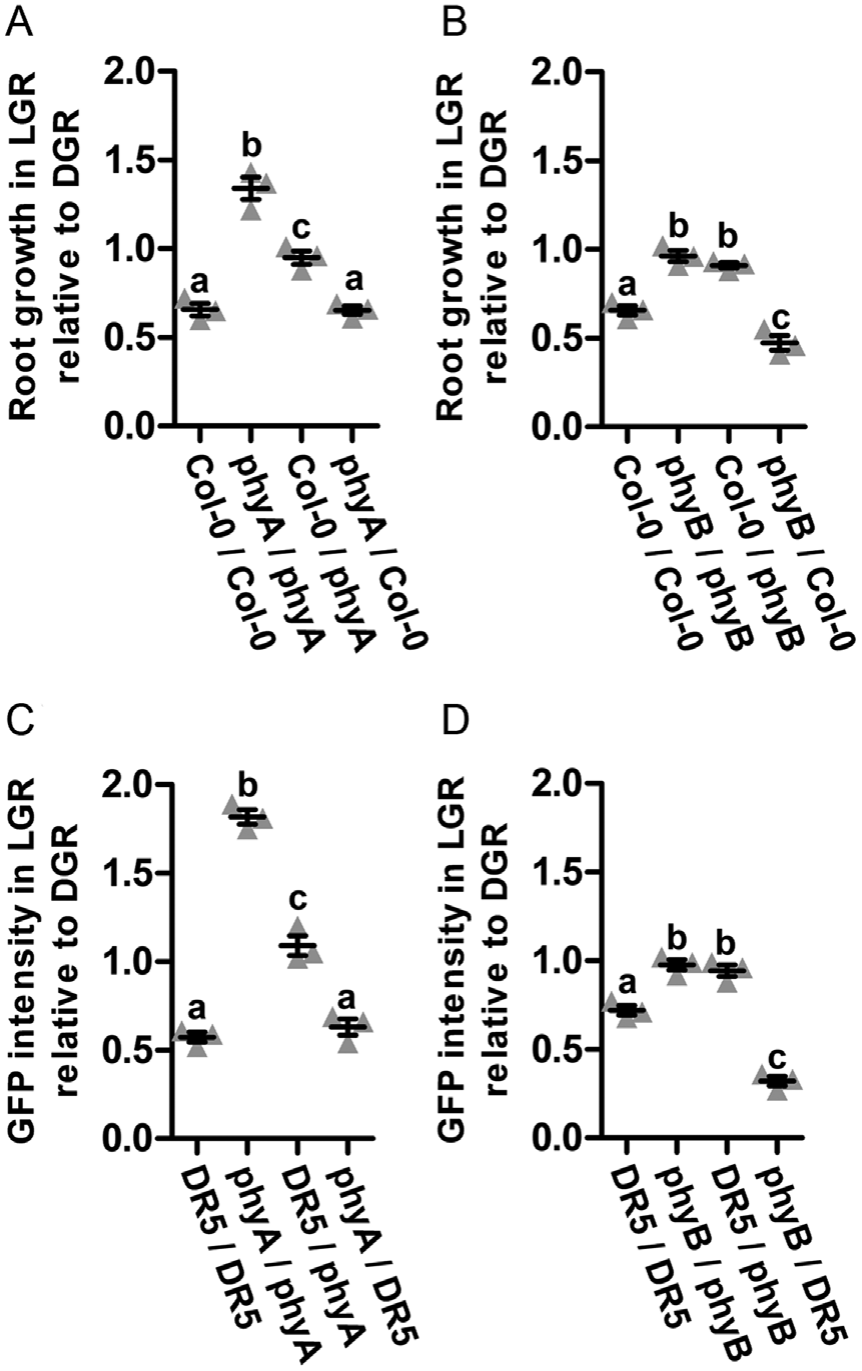
Grafting: local PHYA and PHYB trigger root growth inhibition in response to light. (A, B) Quantification of the root growth of *phyA* and wild-type (Col-0) grafts (A), or *phyB* and Col-0 grafts (B) in the LGR condition, relative to the DGR condition, at 5 d post-grafting. (C, D) Quantification of the corrected total fluorescence (CTF) of *pDR5::GFP* in the root apical meristem (RAM) of indicated grafts at 5 d post-grafting in the LGR relative to the DGR condition. Scion–rootstock combinations were grafted using 4-day-old *phyA* and Col-0 (A), *phyB* and Col-0 (B), *pDR5::GFP* and *phyA × pDR5::GFP* (C), or *pDR5::GFP* and *phyB × pDR5::GFP* (D) seedlings. Graft combinations were compared using a one-way ANOVA followed by Tukey’s test. Lowercase letters indicate statistically different values, *P*<0.05. In the graphs, the horizontal line indicates the mean, error bars represent standard error of the mean, and triangles indicate values of biologically independent observations (*n*=5). Similar results were obtained from two independent experiments.

Since the differential auxin levels in LGR and DGR seedlings must be initiated by detection of light, we next investigated the LGR response in mutants of the three main photoreceptor families in land plants: the R/FR-inducible PHYs, and the blue light-induced CRYs and phototropins PHOTs. Although their main functions might be above-ground, these photoreceptors are also expressed in roots (Van Gelderen *et al.*, 2018). For most of the single *phy*, *cry*, and *phot* mutants, light-grown roots were significantly shorter than dark-grown roots, indicating that the response of root growth to light was not affected (Supplementary Fig. S3B). For the *phyA* and *phyB* mutants, however, LGR and DGR roots were of the same length, suggesting that the sensitivity of the roots to light was lost in these mutants (Fig. 3A, B). Moreover, analysis of the *phyAphyB* double mutant showed a similar loss of light sensitivity. To monitor the response to light quality, Arabidopsis seedlings were grown with their roots covered by clear (LGR), red (RGR), or blue (BGR) translucent plastic, or black paper covers (DGR). Primary root growth was significantly inhibited in LGR and RGR seedlings, but not in BGR or DGR seedlings (Fig. 3B), confirming that inhibition of root growth is specific for R and FR light. The *pDR5::GFP* reporter showed a similar auxin response in the LGR and DGR condition in both the *phyA* and *phyB* mutant background (Fig. 3C, D). In addition, we used quantitative RT-PCR to compare the LGR/DGR expression ratio between wild type and *phy* mutants. The LGR/DGR expression ratio of *YUC4* was significantly increased in the RAM of *phyA* seedlings, compared with the ratio in the RAM of Col-0 and *phyB* seedlings (Fig. 3E). Moreover, the LGR/DGR expression ratio of *YUC6* was significantly increased in *phyA* and *phyB* seedlings, compared with Col-0 seedlings (Fig. 3F). Together, these data suggest that inhibition of *YUC4* and *YUC6* expression by light is regulated by PHYA and partially by PHYB. Finally, to confirm root-localized PHYA and PHYB activation, we performed a series of grafting experiments. The following scion–rootstock combinations were included: wild type–wild type (positive control), mutant–mutant (negative control), wild type–mutant (to study photoactivation in the shoot), and mutant–wild type (to study photoactivation in the root) (Supplementary Fig. S4). As expected, the positive control grafts showed sensitivity to light, and the negative control grafts were insensitive. For both mutants, the grafts with wild-type roots retained light sensitivity, whereas the grafts with mutant roots had lost light sensitivity (Fig. 4A, B), confirming that root-localized photoactivation of PHYA or PHYB initiates root growth inhibition by light. Finally, grafting of *phy × pDR5::GFP* seedlings with wild-type *pDR5::GFP* seedlings confirmed the correlation between primary root growth and auxin response in the RAM of grafted seedlings (Fig. 4C, D). Altogether, the experiments described above showed that FR and R light directly activate root-localized PHYA and PHYB, respectively, to inhibit *YUC4* (PHYA) and *YUC6* (PHYA and PHYB) expression, thus lowering local auxin levels to reduce primary root growth.

### Light-activated root-localized phytochromes repress local auxin biosynthesis via PIF1 and PIF4

**Fig. 5.**
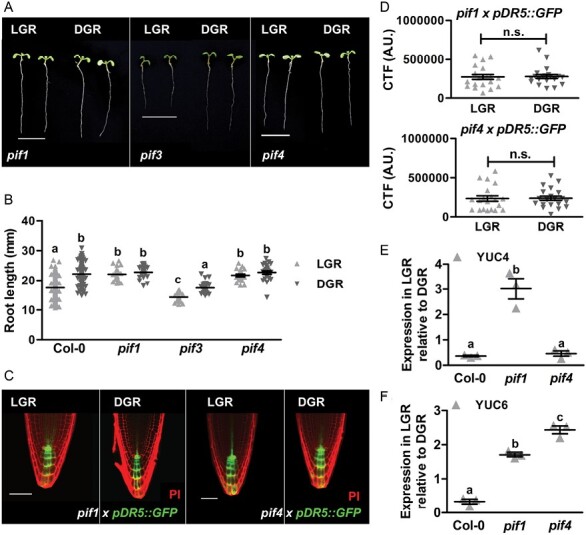
Light represses local auxin biosynthesis through degradation of PIF1 and PIF4. (A) Representative 7-day-old *pif* mutant seedlings grown in the LGR or the DGR condition. For presentation purposes, seedlings were transferred to black agarose plates before photographing. (B) Quantification of the primary root length of 7-day-old Col-0 and *pif* seedlings grown in the LGR or DGR condition. (C) Confocal images of the root apical meristem (RAM) of *pif1 × pDR5::GFP* and *pif4 × pDR5::GFP* (green signal) seedlings grown in the LGR or DGR condition. Root tips were stained with propidium iodide (PI, red signal). (D) Quantification of the corrected total fluorescence (CTF) of *pDR5::GFP* in the RAM. (E, F) Quantitative RT-PCR analysis of *YUC4* (E) and *YUC6* (F) expression in the RAM of 7-day-old Col-0, *pif1*, and *pif4* seedlings grown in the LGR condition relative to the DGR condition. In (B, E, F) primary root lengths were compared using a one-way ANOVA followed by Tukey’s test. Lowercase letters indicate statistically different values, *P*<0.05. In (D) the LGR condition was compared with the DGR condition using a two-sided Student’s *t*-test (n.s., not significant). Scale bars indicate 1 cm in (A) and 50 µm in (C). In (B) (*n*=30), (D) (*n*=20) and (E, F) (*n*=3), the horizontal line indicates the mean, error bars represent standard error of the mean (for some not visible due to limited variation), and triangles indicate values of biologically independent observations. Similar results were obtained from three (A, B and E, F), or from two (C, D) independent experiments.

Photoactivated PHYs can affect gene expression either through inhibition of ubiquitin E3 ligases, such as COP1/SPA, or by inhibition of the basic helix–loop–helix (bHLH) family of PIF transcription factors (Pham *et al.*, 2018; Podolec and Ulm, 2018). Since PIF inhibition is exclusive for PHYA and PHYB signalling, we investigated PIF1 and PIF3, as they are targeted by both photoreceptors, and the PHYB-exclusive target PIF4 for its known role in regulation of auxin biosynthesis (Franklin *et al.*, 2011; Pham *et al.*, 2018). In addition, PIF1 and PIF4 have been shown to bind the *YUC6* promoter, while the *YUC4* promoter region also contains predicted PIF binding sites (Li *et al.*, 2022; Supplementary Fig. S5). Primary root growth measurements of *pif1*, *pif3*, and *pif4* mutants grown in the LGR and DGR condition revealed that *pif1* and *pif4* seedlings were insensitive to root illumination, whereas *pif3* responded similar to wild-type seedlings (Fig. 5A, B). In line with our results in *phyA* and *phyB* mutants, the *pDR5::GFP* response in *pif1* and *pif4* mutants was the same in LGR and DGR conditions (Fig. 5C, D). Moreover, quantitative RT-PCR analysis showed a significant increase in the LGR/DGR expression ratio of *YUC4* in the RAM of *pif1* seedlings, compared with Col-0 and *pif4* seedlings (Fig. 5E). The LGR/DGR expression ratio of *YUC6* was significantly increased in the RAMs of *pif1* and *pif4* seedlings, compared with the ratio in Col-0 seedlings (Fig. 5F). Since the LGR/DGR expression ratios of *YUC4* and *YUC6* in *pif1* mutants were similar to those in *phyA* mutants (Fig. 3E, F), PIF1 is most likely targeted by PHYA in response to FR light exposure of roots. Likewise, the RAMs of *pif4* mutants showed similar LGR/DGR expression ratios of *YUC4* and *YUC6* to *phyB* mutants (Fig. 3E, F), suggesting that PHYB inhibits PIF4 in response to illumination of roots with R light.

### Light-induced inhibition of root growth is partially conserved between Arabidopsis and tomato

**Fig. 6.**
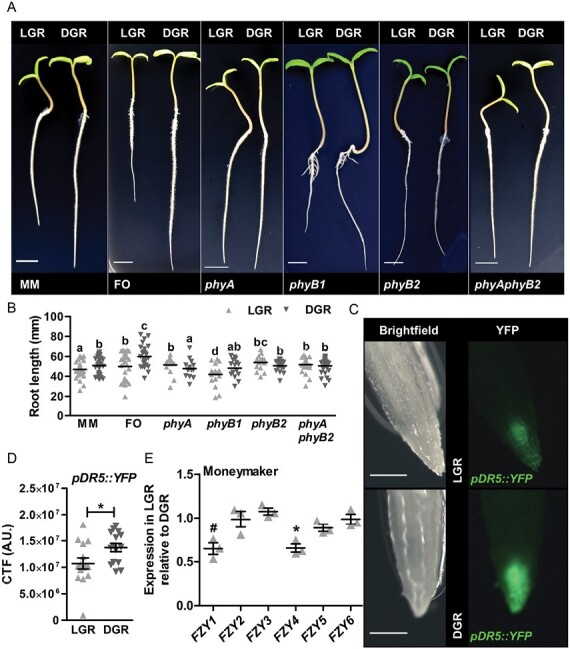
Light-induced inhibition of root growth is (partially) conserved between Arabidopsis and tomato. (A) Representative 5-day-old tomato seedlings of wild-type cultivars Moneymaker (MM) and Foundation (FO), and of *phy* mutants (in MM background) grown in the LGR or the DGR condition. For presentation purposes, seedlings were transferred to black agarose plates before photographing. (B) Quantification of the primary root length of 5-day-old MM, FO, and *phy* seedlings grown in the LGR or DGR condition. (C) Stereo-fluorescence images of the root apical meristem (RAM) of *pDR5::YFP* tomato (M82) seedlings grown in the LGR or DGR condition. (D) Quantification of the corrected total fluorescence (CTF) of *pDR5::YFP* in the RAM. (E) Quantitative RT-PCR analysis of expression of *AtYUC* orthologues *SlFZY1* to *SlFZY6* in the RAM of 5-day-old MM tomato seedlings grown in the LGR condition, relative to the DGR condition. Primary root lengths in (B) were compared using a one-way ANOVA followed by Tukey’s test. Lowercase letters indicate statistically different values, *P*<0.05. In (D, E) the LGR condition was compared with the DGR condition using a two-sided Student’s *t*-test (*P<0.05; #close to significant, *P*=0.08). Scale bars indicate 1 cm in (A) and 0.5 mm in (C). In (B) (*n*=30), (D) (*n*=20) and (E) (*n*=3), the horizontal line indicates the mean, error bars represent standard error of the mean (for some not visible due to limited variation), and triangles indicate values of biologically independent observations. Similar results were obtained from three (A, B, and E), or from two (C, D) independent experiments.

The above results reveal a mechanism that, when conserved in other plant species, might be relevant for optimizing the growth of horticultural crop species, such as tomato. We therefore grew wild-type tomato seedlings of the genotypes Moneymaker (MM) and the commercial hybrid Foundation (FO) in the LGR and DGR conditions. Similar to Arabidopsis, MM and FO seedlings showed a significant reduction in primary root growth in the LGR condition, compared with the DGR condition (Fig. 6A, B). Analysis of MM *phy* mutants in the LGR and DGR condition, showed that both *phyB2* single and *phyAphyB2* double mutant seedling roots were insensitive to light, whereas *phyB1* roots responded the same as wild-type roots. Interestingly, tomato *phyA* roots were significantly longer in the LGR condition, compared with the DGR condition, which is not the case for Arabidopsis *phyA* roots (Fig. 6A, B). As in Arabidopsis, the tomato *pDR5::YFP* reporter line showed that the auxin response in the RAM was significantly reduced in the LGR condition compared with the DGR condition (Fig. 6C, D). Next, we examined gene expression of the tomato auxin biosynthesis genes *SlFZY1-6*. Although described as a functional orthologue of *AtYUC6* (Expósito-Rodríguez *et al.*, 2011), *SlFZY2* expression was similar in both light conditions, indicating that this gene is not inhibited in response to direct root illumination (Fig. 6E). For the *AtYUC4* orthologue, *SlFZY1* (Expósito-Rodríguez *et al.*, 2011), a close to significant (*P*=0.08) decrease in the LGR/DGR expression ratio was observed. Moreover, *SlFZY4* showed a significantly lower LGR/DGR expression ratio, indicating that this gene is important for light-induced root growth inhibition in tomato. To summarize, our data suggest that the PHY-triggered and auxin-modulated growth inhibition by light is conserved between Arabidopsis and tomato, but that not all components of the signalling pathway act in the same way or are shared between these two species.

## Discussion

Culturing Arabidopsis seedlings on growth medium in Petri dishes allows for an easy way to study root growth and development. However, the majority of these *in vitro* systems leave the roots exposed to light, which has been shown to negatively affect root growth and development (Yokawa *et al.*, 2014; Moni *et al.*, 2015). Here we aimed to elucidate the mechanism by which direct root illumination affects root growth, thereby clarifying the consequences of using such LGR systems. Direct illumination of roots has been shown to reduce root growth and to influence lateral root emergence and distribution, anthocyanin accumulation, and even flowering time (Sassi *et al.*, 2012; Silva-Navas *et al.*, 2015). Since the effects of root illumination are so diverse, they are more likely to be caused by photoreceptor signalling than by light-induced stresses such as ROS or DNA damage. So far, studies on root-localized photoreceptor signalling have been contradictory. Analysis of root growth in double *cry* and *phot* mutants, alongside blue LED treatments, indicated that inhibition of root growth is likely to be mediated by blue light photoreceptors (Silva-Navas *et al.*, 2015), while experiments with tissue-specific deficiency in PHY chromophores suggested that this phenotype depends on (far-)red-sensitive photoreceptors (Costigan *et al.*, 2011). In this study, we identified PHYA and PHYB as regulators of root growth based on a screen of single photoreceptor mutants. For this reason, we cannot fully exclude some functional redundancy with blue light photoreceptors, as was indicated by Silva-Navas and colleagues (Silva-Navas *et al.*, 2015). However, our experiments with coloured plastic indicated that R and FR, but not blue, light are reducing root growth. Additional grafting experiments confirmed that both root-localized PHYA and PHYB are required for light sensitivity, indicating that these photoreceptors are the main regulators of root growth inhibition in the LGR condition. When we considered downstream signalling components, PIFs seemed the most likely targets, since PIF signalling is exclusive for PHYA and PHYB. Although PIF3 has been shown to induce primary root growth inhibition in Arabidopsis (Bai *et al.*, 2014), *pif3* mutants remained sensitive to the LGR condition, indicating that its function in primary root growth inhibition is initiated in the shoot and not in the root. While both PIF1 and PIF4 have already been shown to bind to the *YUC6* promoter (Li *et al.*, 2022), until now, these interactions have not been linked to the regulation of root growth. Our analysis of the *pDR5::GFP* reporter and quantitative RT-PCR in *pif* mutants showed that, in the DGR condition, PIF1 and PIF4 stimulate local auxin biosynthesis in the RAM by elevating *YUC4* and *YUC6* expression. Since root cells are extremely sensitive to auxin, these slight changes in local auxin concentrations can have great consequences (Thimann, 1937). Our analysis of the *pDR5::GFP* reporter in combination with NAA treatments revealed that endogenous auxin levels in dark-grown roots are close to optimal, whereas in light-grown roots they are greatly reduced, resulting in shorter roots. The close-to-optimal auxin levels in the DGR condition might correlate to previously reported increased sensitivity to indole-3-acetic acid in DGR seedlings (Silva-Navas *et al.*, 2015). With this experiment, we not only showed that inhibition of root growth by light is mediated by auxin, but also demonstrated once more that the LGR *in vitro* system leads to suboptimal root growth.

**Fig. 7.**
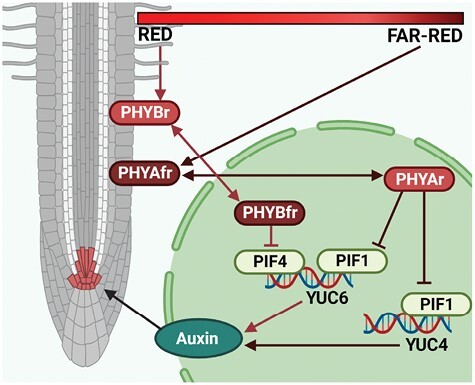
Model for root growth inhibition by local light perception in Arabidopsis roots. Direct illumination of seedling roots with either red (R) or far-red (FR) light inhibits auxin biosynthesis, which ultimately results in decreased primary root growth. In response to FR light, root-localized phytochrome A (PHYA) converts from the inactive PHYAfr state to the active PHYAr state and translocates to the nucleus where it inhibits PHYTOCHROME INTERACTING FACTOR 1 (PIF1). As a result, expression levels of *YUCCA 4* (*YUC4*) and *YUC6* are decreased. Similarly, in response to R light, root-localized PHYB converts from the inactive PHYBr state to the active PHYBfr state and inhibits PIF4 in the nucleus, thereby reducing *YUC6* expression. In both cases this leads to lower auxin levels in the RAM that are suboptimal for root growth. Created with BioRender.com.

Based on our observations in Arabidopsis we propose a model where under natural circumstances, when roots are grown in relative darkness, PIF1 and PIF4 promote expression of *YUC6*, whereas PIF1 also promotes *YUC4* expression (Fig. 7). This results in local auxin biosynthesis in the RAM, and thus in auxin levels that are close to optimal for root growth. However, when roots are exposed to light in widely used *in vitro* systems or aeroponics, PHYA and PHYB photoreceptors are activated. In low R/FR ratios, PHYA converts from the inactive PHYAfr to the active PHYAr conformation that inhibits PIF1, while high R/FR ratios stimulate PHYB to inhibit PIF4. Therefore, all light conditions that expose roots to either R or FR light, or both, will result in PIF inhibition, leading to a decrease in local auxin biosynthesis and a reduced primary root growth. However, light responses observed in the genetic model Arabidopsis do not always translate to an economically important crop such as tomato (Spaninks *et al.*, 2020). By including tomato seedlings, we showed that this mechanism is also present in a horticultural crop, albeit that the components in the signalling pathway are not completely conserved. This implies that the use of aeroponics or light-transmittable substrates could lead to suboptimal root growth in crops, which could result in decreased tolerance to a range of abiotic stresses (Koevoets *et al.*, 2016). On the other hand, additional research into the root response to different spectral qualities might provide us with new ways to steer root architecture towards better crop performance such as a higher yield (Alaguero-Cordovilla *et al.*, 2018). Moreover, root illumination can influence flowering time in Arabidopsis (Silva-Navas *et al.*, 2015), and thus may be used to modulate other important traits to improve crop performance. Finally, the big question that remains to be answered is why plants have developed this molecular mechanism in response to root illumination. Since roots are actively stimulated to grow into the soil via positive gravitropism and negative phototropism (Harmer and Brooks, 2018), it is not entirely surprising that roots develop better in relative darkness. But why would plants actively inhibit root growth when exposed to light? Perhaps root inhibition by light somehow relates to negative phototropism. Previous studies have shown that light affects root halotropism and the gravitropic response, indicating its importance in tropisms (Yokawa *et al.*, 2011; Silva-Navas *et al.*, 2015). Although negative phototropism is primarily regulated by the blue light receptor PHOT1, it has been suggested that PHYA interacts with PHOT1 during root phototropism, possibly by modulating its intracellular distribution, or through induction of PHYTOCHROME KINASE SUBSTRATE 1 (Boccalandro *et al.*, 2008; Han *et al.*, 2008). Moreover, a PHYA-mediated auxin decrease in the RAM of LGR seedling might aid in establishing the auxin gradient that is required for bending during photo- or gravitropic responses. This, however, does not explain the PHYB response in the LGR condition. Aside from its role in light signalling, PHYB is a known thermosensor that, together with PIF4, embodies the main signalling hub in regulation of temperature responses (Casal and Balasubramanian, 2019). Since exposure of roots is likely to simultaneously raise the root temperature, the PHYB responses to light and temperature might be interconnected. Substantial increases in root temperature result in decreased nutrient uptake, enhanced respiration, and overall growth inhibition (Du and Tachibana, 1994). A light-induced decrease in RAM size could contribute to, or be a result of, root cell respiration induced by high temperature. In addition, PHYB–PIF4 signalling regulates auxin biosynthesis in hypocotyls in response to heat stress (Sun *et al.*, 2012), suggesting the possibility that, in roots, light and temperature co-regulate auxin levels via PHYB–PIF4 to guarantee sufficient water and nutrient uptake.

## Supplementary data

The following supplementary data are available at *JXB* online.

Fig. S1. LGR and DGR growth conditions.

Fig. S2. Arabidopsis DGR seedlings show a reduced shoot/root ratio despite their longer hypocotyls.

Fig. S3. Seedling roots of several Arabidopsis photoreceptor mutants are shorter in light-grown conditions.

Fig. S4. Photographs of grafted seedlings.

Fig. S5. Predicted binding sites in the YUC4 promoter region.

Table S1. Plant lines used in this study.

Table S2. Primers used in this study.

Table S3. CAPS/ PCR-RFLP markers for genotyping.

Table S4. Linear regression analysis.

erad163_suppl_Supplementary_Tables_S1-S4_Figures_S1-S5

## Data Availability

The raw data that support the findings presented in this study are available from the corresponding author (RO) upon request.
