# Supplementary material for: Local phytochrome signalling limits root growth in light by repressing auxin biosynthesis
Source: J Exp Bot. 2023 May 4;74(15):4642–53. doi: 10.1093/jxb/erad163 (PMC10433924; doi:10.1093/jxb/erad163)
Supplement: erad163_suppl_Supplementary_Tables_S1-S4_Figures_S1-S5 [file erad163_suppl_supplementary_tables_s1-s4_figures_s1-s5.pdf]

Supplementary data (Tables S1-S4 and Figures S1-S5)

Table S1: Plant lines used in this study.

Arabidopsis mutant lines were obtained from Nottingham Arabidopsis Stock Centre (NASC). Tomato mutant lines were obtained from Tomato Genetics Resource Centre (TGRC).

Plant line	Description	Source	Reference
**Arabidopsis**			
Columbia (Col-0)	Natural Arabidopsis accession	-	Redei, 1992
Landsberg *erecta* (L*er*)	Natural Arabidopsis accession	-	Redei, 1992
Moneymaker (MM)	Standard non-hybrid cultivar	Nunhems	-
Foundation (FO)	Commercial hybrid	Nunhems	-
yuc4 (SM_3_16128)	Transposon insertion in exon of At5g11320	-	Cheng et al., 2006
yuc6 (SALK_093708)	T-DNA insertion in intron of At5g25620	-	Cheng et al., 2006
phyA (SALK_014575)	T-DNA insertion in exon of At1g09570	NASC	Ruckle et al., 2007
phyB (SALK_ 022035)	T-DNA insertion in exon of At2g18790	NASC	Mayfield et al., 2007
phyC (phyC-3)	3 kbp deletion in At5g35840	NASC	Monte et al., 2003
phyD (SALK_027956)	T-DNA insertion in exon of At4g16250	NASC	Christians et al., 2012
phyE (SALK_092529)	T-DNA insertion in exon of At4g18130	NASC	Warnasooriya et al., 2011
cry1 (SALK_069292)	T-DNA insertion in exon of At4g08920	NASC	Ruckle et al., 2007
cry2 (cry2-1)	Large deletion (2/3) in At1g04400	NASC	Guo et al., 1998
phot1 (SAIL_1232_C01)	T-DNA insertion in exon of At3g45780	NASC	McElver et al., 2001
phot2 (SALK_142275)	T-DNA insertion in exon of At5g58140	NASC	Ruckle et al., 2007
phyAphyB (phyA-201 phyB-5)	Substitution Q980STOP in At1g09570 x substitution W552STOP in At2g18790	NASC	Reed et al., 1994
pif1 (SAIL_256_G07)	T-DNA insertion in exon of At2g20180	NASC	Stephenson et al., 2009
pif3 (SALK_030753)	T-DNA insertion in intron of At1g09530	NASC	Kim et al., 2003
pif4 (SAIL_1288_E07)	T-DNA insertion in intron of At2g43010	NASC	Leivar et al., 2008a
pif1pif3pif4 (SAIL_256_G01, pif3-3, SAIL_1288_E07)	2.5 kbp deletion in At1g09530 x T-DNA insertion in At2g20180 and At2g43010	NASC	Leivar et al., 2008b
pin1 (SALK_047613)	T-DNA insertion in exon of At1g73590	NASC	Smith et al., 2006
pin2 (*eir1-1*)	Diepoxybutane mutation in exon of At5g57090	NASC	Guenot et al., 2012
pin4-3	Transposon insertion in exon of At2g01420	NASC	Guenot et al., 2012
pin7-2	T-DNA insertion in exon of At1g23080	NASC	Guenot et al., 2012
auxlaxq (*aux1-21, lax1, lax2, lax3)*	EMS mutation in exon of At2g38120 x T-DNA insertion in exon of At5g01240 x T-DNA insertion in exon of At2g21050 x T-DNA insertion in exon of At1g77690	NASC	Bainbridge et al., 2008
pDR5::GFP	Synthetic auxin-responsive reporter (Col-0)	-	Ottenschlager et al., 2003
phyA x pDR5::GFP	SALK_014575 crossed with DR5 reporter	-	This study
phyB x pDR5::GFP	SALK_022035 crossed with DR5 reporter	-	This study
pif1 x pDR5::GFP	SAIL_256_G07 crossed with DR5 reporter	-	This study
pif4 x pDR5::GFP	SAIL_1288_E07 crossed with DR5 reporter	-	This study
**Tomato**			
phyA (phyA-1)	Null-mutant (fri^1)	TGRC	Van Tuinen et al., 1995a
phyB1 (phyB1-1)	Null-mutant (tri^1)	TGRC	Van Tuinen et al., 1995b
phyB2 (phyB2-1)	Null-mutant (70F)	TGRC	Weller et al., 2000
phyAphyB2	Null-mutant (fri^1) x Null mutant (70F)	TGRC	Weller et al., 2000
pDR5::YFP	Synthetic auxin-responsive reporter (M82)	Kuhlemeier	Ben-Gera et al., 2012

Table S2: Primers used in this study.

Primer name	Target gene	Sequence 5'→3'	Experiment
LB1 (SAIL T-DNA)	N/A	GCCTTTTCAGAAATGGATAAATA	Genotyping
LBb1.3 (SALK T-DNA)	N/A	ATTTTGCCGATTTCGGAAC	Genotyping
Border seq (SM transposon)	N/A	TACGAATAAGAGCGTCCATTTTAGAGTGA	Genotyping
SALK_014575 (*phyA*) FW	At1g09570	CCAGTCAGCTCAGCAATTTTC	Genotyping
SALK_014575 (*phyA*) RV	At1g09570	AATGCAAAACATGCTAGGGTG	Genotyping
SALK_ 022035 (*phyB*) FW	At2g18790	CATCATCAGCATCATGTCACC	Genotyping
SALK_ 022035 (*phyB*) RV	At2g18790	TTCACGAAGGCAAAAGAGTTG	Genotyping
SM_3_16128 (*yuc4*) FW	At5g11320	CCCTTCTTAGACCTACTCTAC	Genotyping
SM_3_16128 (*yuc4*) RV	At5g11320	GCCCAACGTAGAATTAGCAAG	Genotyping
SALK_093708 (*yuc6*) FW	At5g25620	CCAGCCTTTGTATTTTCCCGT	Genotyping
SALK_093708 (*yuc6*) RV	At5g25620	CCGGAAAAAGGGTTCTTGTCG	Genotyping
phyA-201 (double mutant) FW	At1g09570	GAAGTGTTGACTGCTTCCACGAGT	Genotyping
phyA-201 (double mutant) RV	At1g09570	TAGCAAGATGCACAGAACGCC	Genotyping
phyB-5 (double mutant) FW	At2g18790	CGTGACGCGCCTGCTGGAATTGTT	Genotyping
phyB-5 (double mutant) RV	At2g18790	TCCATTGATGCAGCCTCCGGCA	Genotyping
phyC-3 FW	At5g35840	ATGTCATCGAACACTTCACG	Genotyping
phyC-3 RV	At5g35840	TCAAATCAAGGGAAATTCTG	Genotyping
SALK_027956 (*phyD*) FW	At4g16250	AACCCGGTAGAATCAGAATGG	Genotyping
SALK_027956 (*phyD*) RV	At4g16250	ATCGGTTACAGTGAAAATGCG	Genotyping
SALK_092529 (*phyE*) FW	At4g18130	AAAGAGGCGGTCTAGTTCAGC	Genotyping
SALK_092529 (*phyE*) RV	At4g18130	TATCAGTGGTTAAACCCGTCG	Genotyping
SALK_069292 (*cry1*) FW	At4g08920	TTCATGCCACTTGGTTAGACC	Genotyping
SALK_069292 (*cry1*) RV	At4g08920	TCCCGACAGACTGGATACATC	Genotyping
cry2-1 FW	At1g04400	ATGAAGATGGACAAAAAGAC	Genotyping
cry2-1 RV	At1g04400	TCATTTGCAACCATTTTTTC	Genotyping
SAIL_1232_C01 (*phot1*) FW	At3g45780	ACATAGGATGCAGCAGAAACG	Genotyping
SAIL_1232_C01 (*phot1*) RV	At3g45780	CAGTAGACTGGTGGGCTCTTG	Genotyping
SALK_142275 (*phot2*) FW	At5g58140	TCCATCTCCTTTGAATGATGC	Genotyping
SALK_142275 (*phot2*) RV	At5g58140	AGTGTCATTGCTCACGGATTC	Genotyping
phyA-1 FW	Solyc10g044670	TAACTGAATACACCATTCCCTTAACC	Genotyping
phyA-1 RV	Solyc10g044670	ATAATCGCTCTATAGTCACC	Genotyping
phyB1-1 FW	Solyc01g059870	CTAAAATTCAAAGAGGAGGTCAGATT	Genotyping
phyB1-1 RV	Solyc01g059870	GAAGGGGTAAAAAGGGTCCTAA	Genotyping
phyB2-1 FW	Solyc05g053410	GACGAGTAACATTCACATGA	Genotyping
phyB2-1 RV	Solyc05g053410	GCTTAGGCAACACTAGGTTA	Genotyping
SAIL_256_G07 (*pif1*) FW	At2g20180	AAGGAAGGAGGAGGAATAGGC	Genotyping
SAIL_256_G07 (*pif1*) RV	At2g20180	CATGAATTTCTCGAGGCTGAG	Genotyping
SALK_030753 (*pif3*) FW	At1g09530	AGTCTGTTGCTTCTGCTACGC	Genotyping
SALK_030753 (*pif3*) RV	At1g09530	TTGCATAAGGCATTCCCATAC	Genotyping
SAIL_1288_E07 (*pif4*) FW	At2g43010	AATACATTTTGCAGGCAATCG	Genotyping
SAIL_1288_E07 (*pif4*) RV	At2g43010	CGTAATGAAGTTGCACGTTTACTC	Genotyping
pif3-3 WT (triple mutant) FW	At1g09530	AGAAGCAATTTGGTCACCATGCTC	Genotyping
pif3-3 WT (triple mutant) RV	At1g09530	TGCATACAAATAGTCGATCGTATG	Genotyping
pif3-3 DEL (triple mutant) FW	At1g09530	GGTGTGTATGTGAGAAGGTACATCCATCG	Genotyping
pif3-3 DEL (triple mutant) RV	At1g09530	AAGCTTAGCTTTGGTGAGCCTGAAAAGCT	Genotyping
SALK_047613 (*pin1*) FW	At1g73590	TTCCATAAAGTCATGATTAAGCACA	Genotyping
SALK_047613 (*pin1*) RV	At1g73590	CGGTGGGAACAACATAAGCAA	Genotyping
eir1-1 (*pin2*) FW	At5g57090	GGTACCAAATGATCACCGGCAAAGACAT	Genotyping
eir1-1 (*pin2*) RV	At5g57090	GAAGAGATCATTGATGAGGC	Genotyping
pin4-3 FW	At2g01420	CAACGCCGTTAAATATGG	Genotyping
pin4-3 RV	At2g01420	TGCAGCAAAACCCACACTTTTACTTC	Genotyping
pin7-2 FW	At1g23080	TTTACTTGAACAATGGCCACAC	Genotyping
pin7-2 RV	At1g23080	GGTAAAGGAAGTGCCTAACGG	Genotyping
aux1-21 FW	At2g38120	TGCTACCAAAGCACTACTACTAC	Genotyping
aux1-21 RV	At2g38120	GAAATGGCTGAAACCAACTCAA	Genotyping
lax1 FW	At5g01240	ATATGGTTGCAGGTGGCACA	Genotyping
lax1 RV	At5g01240	GTAACCGGCAAAAGCTGCA	Genotyping
lax2 FW	At2g21050	ATGGAGAACGGTGAGAAAGCAGC	Genotyping

lax2 RV	At2g21050	CGCAGAAGGCAGCGTTAGCG	Genotyping
lax3 FW	At1g77690	TACTTCACCGGAGCCACCA	Genotyping
lax3 RV	At1g77690	TGATTGGTCCGAAAAAGG	Genotyping
PP2A-3 FW	At2g42500	ACGTGGCCAAAATGATGCAA	qRT-PCR
PP2A-3 RV	At2g42500	TCATGTTCTCCACAACCGCT	qRT-PCR
β-tubulin-6 FW	At5g12250	TGGGAACTCTGCTCATATCT	qRT-PCR
β-tubulin-6 RV	At5g12250	GAAAGGAATGAGGTTCACTG	qRT-PCR
YUCCA1 FW	At4g32540	TTAGCTTAGACCTCGTCGGACAT	qRT-PCR
YUCCA1 RV	At4g32540	TGGCAACACATGAACGGTGT	qRT-PCR
YUCCA2 FW	At4g13260	TGTTTTGGACGTTGGCACTCT	qRT-PCR
YUCCA2 RV	At4g13260	TACCCGTTTCAACTCCGGATA	qRT-PCR
YUCCA3 FW	At1g04610	CCTACGCAGCCAACTTTGACA	qRT-PCR
YUCCA3 RV	At1g04610	GCCCGAACGTCTCATCATATTT	qRT-PCR
YUCCA4 FW	At5g11320	TCTAGCCGTAGCGGCTTGTTT	qRT-PCR
YUCCA4 RV	At5g11320	AAACAATCGGTTCTCTCGAGGA	qRT-PCR
YUCCA5 FW	At5g43890	TGTCCAGTCTGCTCGATACGA	qRT-PCR
YUCCA5 RV	At5g43890	CACCGGCAGATATATTCCATCTC	qRT-PCR
YUCCA6 FW	At5g25620	CGGTATGGAGGTTTGTTTGGAT	qRT-PCR
YUCCA6 RV	At5g25620	ATGGACAGCCCAAAAGTTGAAG	qRT-PCR
YUCCA7 FW	At2g33230	CCCGGAGTATCCAACGAAGTAC	qRT-PCR
YUCCA7 RV	At2g33230	TGATTGGACCGTCTCATTGAAC	qRT-PCR
YUCCA8 FW	At4g28720	TGACCTAGCAAACCATTTCGCT	qRT-PCR
YUCCA8 RV	At4g28720	CATCTTCATTGCAAGCTCAAACG	qRT-PCR
YUCCA9 FW	At1g04180	TTCTCAGAGCGGCGATGTGT	qRT-PCR
YUCCA9 RV	At1g04180	CACAACGAATGGGACTCCTTGA	qRT-PCR
YUCCA10 FW	At1g48910	AAGTATGCTCCAGTGGCGATG	qRT-PCR
YUCCA10 RV	At1g48910	GGAAGAGTCCGTACTTGGAGAGATC	qRT-PCR
YUCCA11 FW	At1g21430	GACGAATACGCCACACGTTTC	qRT-PCR
YUCCA11 RV	At1g21430	ACCATCTTTGAAGTACGCGGA	qRT-PCR
TAA1 FW	At1g70560	GCAGAGCTGGAGAGCGTTGTG	qRT-PCR
TAA1 RV	At1g70560	CTTCATGTTGGCGAGTCTCTCGAG	qRT-PCR
TAR1 FW	At1g23320	CAGGAAGGCTCCTCAGACATTGC	qRT-PCR
TAR1 RV	At1g23320	CGCTGGTCAGAGTTATGAGACACC	qRT-PCR
TAR2 FW	At4g24670	GGTTGTGTCAGACAGTTGTGGG	qRT-PCR
TAR2 RV	At4g24670	GGTTGTGGCTCAAAGACCCTGC	qRT-PCR
TIP41 FW	Solyc10g049850	ATGGAGTTTTTGAGTCTTCTGC	qRT-PCR
TIP41 RV	Solyc10g049850	GCTGCGTTTCTGGCTTAGG	qRT-PCR
SAND FW	Solyc03g115810	TTGCTTGGAGGAACAGACG	qRT-PCR
SAND RV	Solyc03g115810	GCAAACAGAACCCCTGAATC	qRT-PCR
SlFZY1 FW	Solyc06g065630	GTACTCGACGTTGGAGCATTATC	qRT-PCR
SlFZY1 RV	Solyc06g065630	TGAAGAAATCATTTCCCTTAAACC	qRT-PCR
SlFZY2 FW	Solyc08g068160	AGGAATGGAGGTGTGTTTGG	qRT-PCR
SlFZY2 RV	Solyc08g068160	GGGACGTGTCACCGAGTAA	qRT-PCR
SlFZY3 FW	Solyc09g091090	TGGAGAAATACAAGAAATTGATTG	qRT-PCR
SlFZY3 RV	Solyc09g091090	AATCCAACTGCATATAAACCAGC	qRT-PCR
SlFZY4 FW	Solyc06g008050	TCGATTCTGTTCTTCTTGCTACT	qRT-PCR
SlFZY4 RV	Solyc06g008050	CTGATAGTCCTCTTCTTGTGAAA	qRT-PCR
SlFZY5 FW	Solyc06g083700	GGCACCGTTGAACTTGTCAC	qRT-PCR
SlFZY5 RV	Solyc06g083700	CCTTTCCAATTATTTGGGATTT	qRT-PCR
SlFZY6 FW	Solyc09g074430	CCAGAAGAAGGACCATTTGC	qRT-PCR
SlFZY6 RV	Solyc09g074430	TACCATTTTCCACCACAACATC	qRT-PCR

Table S3: CAPS / PCR-RFLP markers for genotyping

PCR fragment	CAPS / RFLP	Wild-type product	Mutant product
phyA-201	HinfI	±190 bp	241 bp
phyB-5	BsaBI	666 bp	±250 bp
phyA-1	EcoNI	236 bp	±180 bp
phyB1-1	HinfI	±100 bp	193 bp
phyB2-1	FokI	±300 bp	536 bp

Table S4: Linear regression analysis.

Linear regression analysis of the correlation between NAA concentration and *pDR5::GFP* expression in the RAM. *y*-values are the mean values of the dot plot shown in **Figure 2D**. *σ(x,y)* = covariance of *x*- and *y*-values, *σ(x)* = variance of *x*-values, and *σ(y)* = variance of *y*-values. Regression coefficient *a* indicates the y-intercept, and regression coefficient *b* indicates the slope.

Light condition	NAA concentration (*x*)	GFP signal (*y*)	$x_i - x_{mean}$	$y_i - y_{mean}$	*σ(x,y)*	*σ(x)*	*σ(y)*
LGR	0	277979	-25	-151755	3793879	625	23029630610
	10	333101	-15	-96633	1449497	225	9337968900
	20	414728	-5	-15006	75031	25	225185038
	30	454278	5	24544	122719	25	602399755
	40	534336	15	104601	1569027	225	10941543537
	50	563983	25	134249	3356221	625	18022749251
**Mean**	**25**	**429734**	**0**	**0**	**1727729**	**292**	**10359912848**
DGR	0	525449	-8.75	-64573	565011	76.6	4169640043
	5	565031	-3.75	-24991	93715	14.1	624537586
	10	614962	1.25	24940	31175	1.6	622016070
	20	654645	11.25	64623	727012	126.6	4176164441
**Mean**	**8.75**	**590022**	**0**	**0**	**354228**	**54.7**	**2398089535**
	**Correlation coefficient (*r*)**	**Regression coefficient (*a*)**			**Regression coefficient (*b*)**		
LGR	0.993	281643			5924		
DGR	0.978	533345			6477		
	**p-value slope comparison (*b*)**						
LGR vs DGR	0.609						

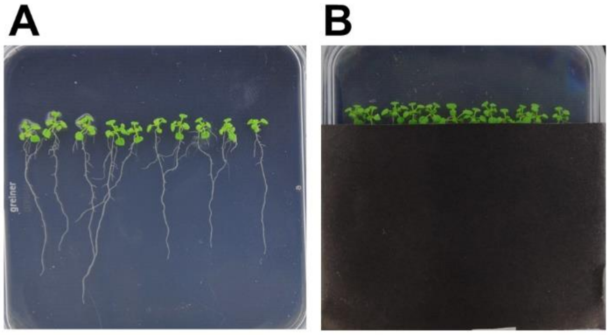

Figure S1: LGR and DGR growth conditions.

A. Arabidopsis **s**eedlings grown in the light-grown root (LGR) condition, where the shoots and roots are exposed to light. **B.** Arabidopsis seedlings grown in the dark-grown (DGR) condition, where only the shoots are exposed to light.

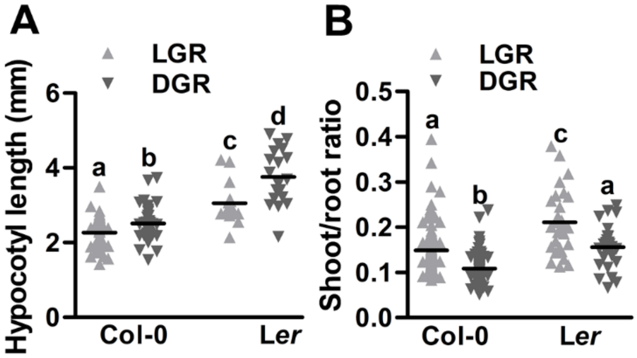

Figure S2: Arabidopsis DGR seedlings show a reduced shoot/root ratio despite their longer hypocotyls.

A. Quantification of the hypocotyl length of 7-day-old Arabidopsis seedings of ecotypes Columbia (Col-0) and Landsberg *erecta* (L*er*) that were grown in light-grown roots (LGR) or dark-grown roots (DGR) conditions. **B.** Quantification of the shoot/root ratio of 7-day-old Col-0 and L*er* seedlings that were grown in LGR or DGR conditions. Hypocotyl lengths or shoot/root ratios were compared using a one-way ANOVA followed by a Tukey's test (letters **a**, **b**, **c**, and **d** indicate statistically different values, $p<0.05$). In the graphs, the horizontal line indicates the mean, error bars indicating standard error of the mean are not visible due to limited variation, and triangles indicate values of biologically independent observations (n=30). Similar results were obtained from three independent experiments.

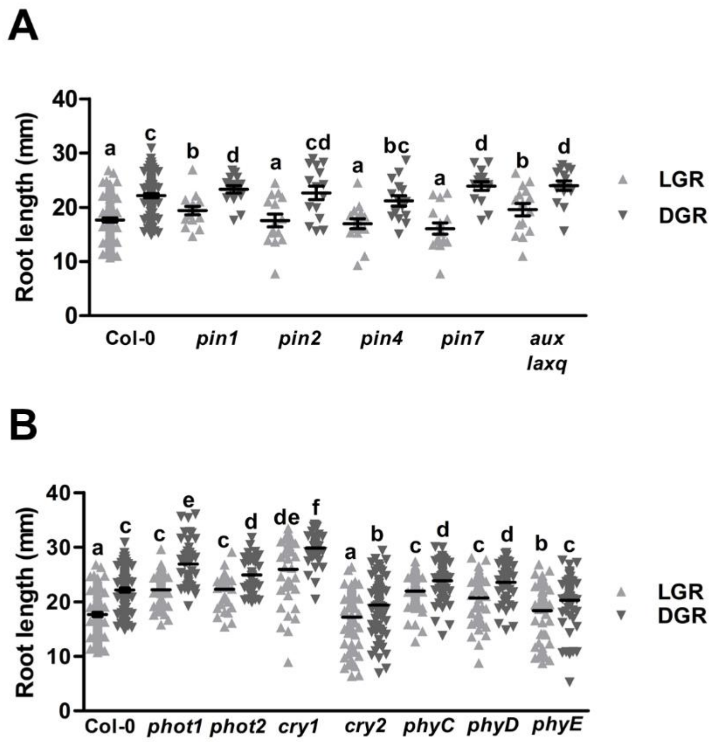

Figure S3: Seedling roots of several Arabidopsis photoreceptor mutants are shorter in light-grown conditions.

A. Quantification of the primary root length of 7-day-old Arabidopsis pin-formed (*pin*) and auxin1/like-aux1 (aux/lax) mutants that were grown in LGR or DGR conditions. **B.** Quantification of the primary root length of 7-day-old Arabidopsis phototropin (*phot*), cryptochrome (*cry*) or phytochrome (*phy*) single mutants that were grown in LGR or DGR conditions. Primary root lengths were compared using a one-way ANOVA followed by a Tukey's test (letters **a**, **b**, **c**, **d**, **e**, and **f** indicate statistically different values, $p<0.05$). In the graphs, the horizontal line indicates the mean, error bars represent standard error of the mean (for some not visible due to limited variation), and triangles indicate values of biologically independent observations (n=30). Similar results were obtained from three independent experiments.

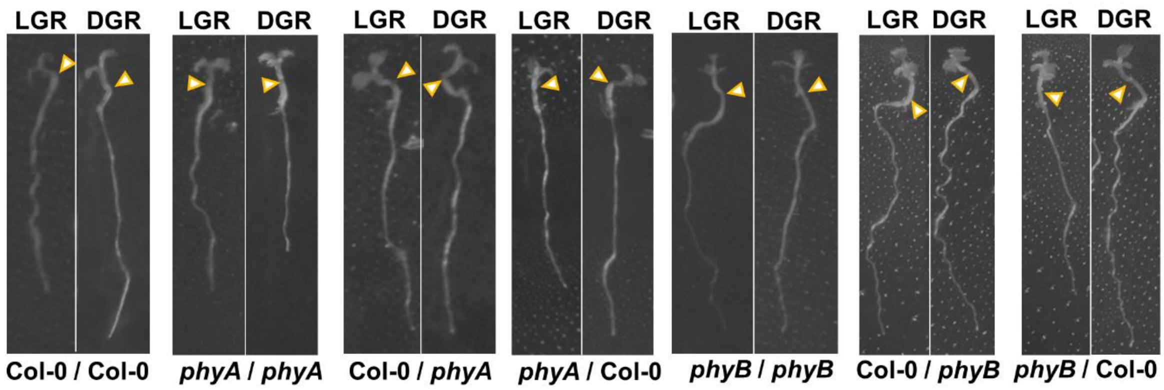

Figure S4: Photographs of grafted seedlings.

4-day-old Arabidopsis **s**eedlings were grafted in the following scion/rootstock combinations: Col-0 / Col-0, *phyA* / *phyA*, Col-0 / *phyA*, *phyA /* Col-0, *phyB / phyB*, Col-0 / *phyB*, and *phyB /* Col-0 (left to right). The graft junction is indicated with a yellow arrowhead. The grafted seedlings were placed in the LGR and DGR growth conditions. At 5 days post-grafting, the seedlings were photographed for root measurements in ImageJ.

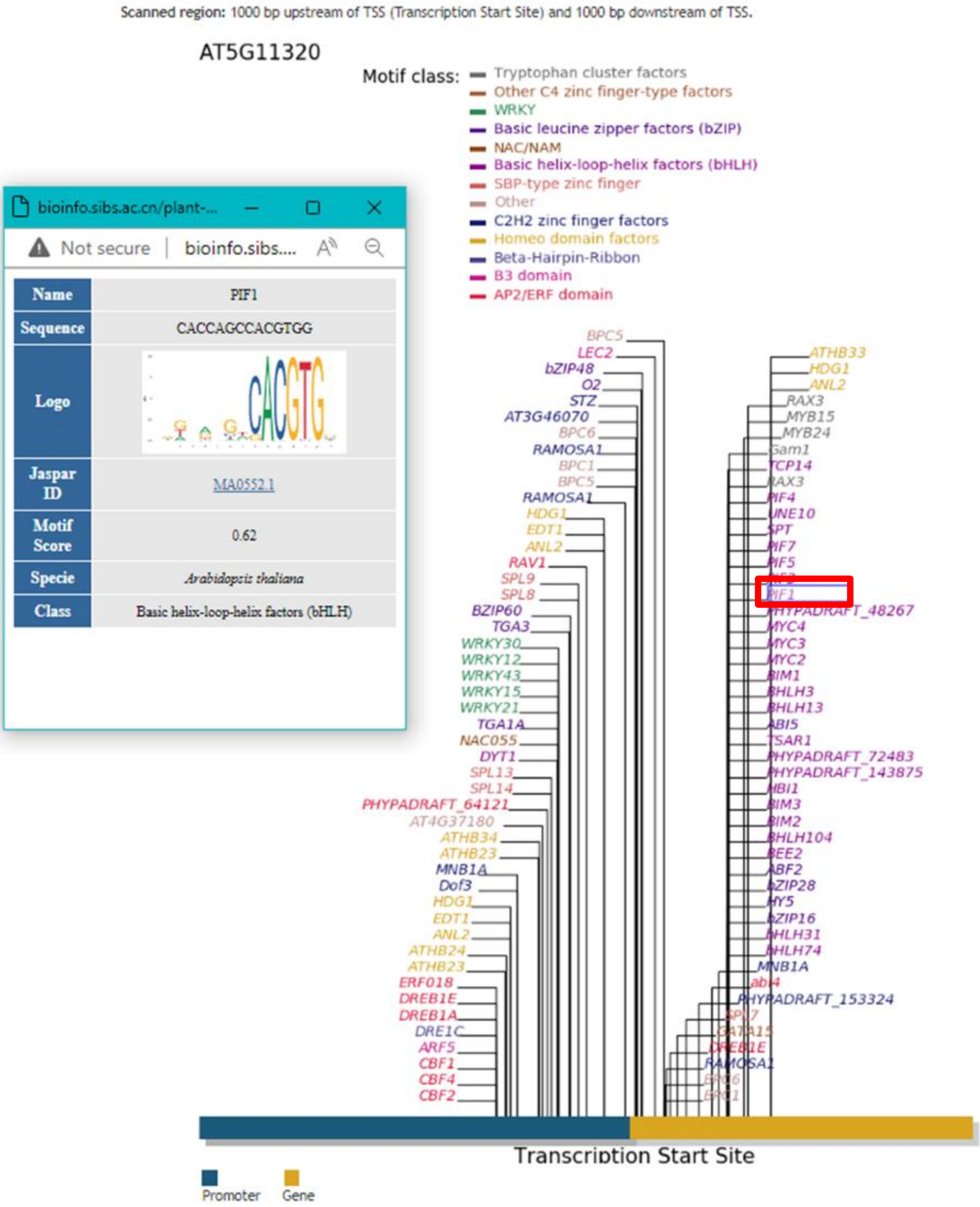

Figure S5: Predicted binding sites in the *YUC4* promoter region.

The Plant regulomics webtool predicts that PIF1, among other PIFs, can bind to the *YUC4* promoter region via an E-box motif (CACGTG).

Supplementary references

Bainbridge, K., Guyomarc'h S., Bayer, E., Swarup, R., Bennet, M., Mandel, T., and Kuhlemeier, C. (2008). Auxin influx carriers stabilize phyllotactic patterning. *Genes & Development* 22, 810-823.

Ben-Gera, H., Shwartz, I., Shao, M. R., Shani, E., Estelle, M., and Ori, N. (2012). ENTIRE and GOBLET promote leaflet development in tomato by modulating auxin response. *Plant Journal.* 70(6), 903-915.

Cheng, Y., Dai, X., and Zhao, Y. (2006**).** Auxin biosynthesis by the YUCCA flavin monooxygenases controls the formation of floral organs and vascular tissues in Arabidopsis. *Genes & Development* 20(13), 1790-1799.

Christians, M. J., Gingerich, D. J., Hua, Z., Lauer, T. D., and Vierstra, R. D. (2012). The light-response BTB1 and BTB2 proteins assemble nuclear ubiquitin ligases that modify phytochrome B and D signaling in Arabidopsis. *Plant Physiology* 160, 118-134.

Guenot, B., Bayer, E., Kierzkowski, D., Smith, R. S., Mandel, T., Zádniková, P., Benková, E., and Kuhlemeier, C. (2012). PIN1-independent leaf initiation in Arabidopsis. *Plant Physiology* 159, 1501-1510.

Guo, H., Yang, H., Mockler, T. C., and Lin, C. (1998). Regulation of flowering time by Arabidopsis photoreceptors. *Science* 279, 1360-1363.

Kim, J., Yi, H., Choi, G., Shin, B., Song, P. S., and Choi, G. (2003). Functional characterization of phytochrome interacting factor 3 in phytochrome-mediated light signal transduction. *The Plant Cell* 15, 2399-2407.

Leivar, P., Monte, E., Al-Sady, B., Carle, C., Storer, A., Alonso, J. M., Ecker, J. R., and Quail, P. H. (2008a). The Arabidopsis phytochrome-interacting factor PIF7, together with PIF3 and PIF4, regulates responses to prolonged red light by modulating phyB levels. *The Plant Cell* 20, 337–352.

Leivar, P., Monte, E., Oka, Y., Liu, T., Carle, C., Castillon, A., Huq, E., and Quail, P. H. (2008b). Multiple phytochrome-interacting bHLH transcription factors repress premature seedling photomorphogenesis in darkness. *Current Biology* 18, 1815-1823.

Mayfield, J. D., Folta, K. M., Paul, A. L., and Ferl, R. J. (2007). The 14-3-3 proteins μ and υ influence transition to flowering and early phytochrome response. *Plant Physiology.* 145, 1692–1702.

McElver, J., Tzafrir, I., Aux, G., Rogers, R., Ashby, C., Smith, K., Thomas, C., Schetter, A., Zhou, Q., Cushman, M. A., Tossberg, J., Nickle, T., Levin, J. Z., Law, M., Meinke, D., and Patton, D. (2001). Insertional mutagenesis of genes required for seed development in *Arabidopsis thaliana*. *Genetics* 159, 1751-1763.

Monte, E., Alonso, J. M., Ecker, J. R., Zhang, Y., Li, X., Young, J., Austin-Phillips, S., and Quail, P. H. (2003). Isolation and characterization of phyC mutants in Arabidopsis reveals complex crosstalk between phytochrome signaling pathways. *The Plant Cell* 15, 1962-1980.

Ottenschlager, I., Wolff, P., Wolverton, C., Bhalerao, R. P., Sandberg, G., Ishikawa, H., Evans, M., and Palme, K. (2003). Gravity-regulated differential auxin transport from columella to lateral root cap cells. *Proceedings of the National Academy of Sciences U. S. A.* 100, 2987-2991.

Redei, G. P. (1992), A heuristic glance at the past of Arabidopsis genetics. *Methods in Arabidopsis Research*, 1-15.

Reed, J. W., Nagatani, A., Elich, T. D., Fagan, M., and Chory, J. (1994). Phytochrome A and phytochrome B have overlapping but distinct functions in Arabidopsis development. *Plant Physiology*. 104, 1139-1149.

Ruckle, M. E., DeMarco, S. M., and Larkin, R. M. (2007). Plastid signals remodel light signaling networks and are essential for efficient chloroplast biogenesis in Arabidopsis. *The Plant Cell* 19, 3944–3960.

Smith, R. S., Guyomarc'h, S., Mandel, T., Reinhardt, D., Kuhlemeier, C., and Prusinkiewicz, P. (2006). A plausible model of phyllotaxis. *Proceedings of the National Academy of Sciences U. S. A.* 103, 1301-1306.

Stephenson, P. G., Fankhauser, C., and Terry, M. J. (2009). PIF3 is a repressor of chloroplast development. *Proceedings of the National Academy of Sciences U. S. A.* 106, 7654–7659.

Van Tuinen, A., Kerckhoffs, L. H. J., Nagatani, A., Kendrick, R. E., and Koornneef, M. (1995a). Far-red light-insensitive, phytochrome A-deficient mutants of tomato. *Molecular & General Genetics* 246, 133-141.

Van Tuinen, A., Kerckhoffs, L. H. J., Nagatani, A., Kendrick, R. E., and Koornneef, M. (1995b). A temporarily red light-insensitive mutant of tomato lacks a light-stable, B-like phytochrome. *Plant Physiology*. 108, 939-947.

Warnasooriya, S. N., Porter, K. J., and Montgomery, B. L. (2011). Tissue- and isoform-specific phytochrome regulation of light-dependent anthocyanin accumulation in *Arabidopsis thaliana*. *Plant Signaling & Behavior*. 6, 624-631.

Weller, J. L., Schreuder, M. E. L., Smith, H., Koornneef, M., and Kendrick, R. E. (2000). Physiological interactions of phytochromes A, B1 and B2 in the control of development in tomato. *The Plant Journal*. 24, 345-356.
